# Diabetes knowledge and its association with the weight status among residents of Jeddah City, Saudi Arabia

**DOI:** 10.1038/s41387-018-0055-8

**Published:** 2018-09-07

**Authors:** Hebah A. Kutbi, Hala H. Mosli, Ahmed H. Alhasan, Rana H. Mosli

**Affiliations:** 1 Department of Clinical Nutrition, Faculty of Applied Medical Sciences, King Abdulaziz University, P.O. Box 80215, Jeddah, 21589 Saudi Arabia; 2 Division of Endocrinology & Metabolism, Department of Medicine, Faculty of Medicine, King Abdulaziz University, Jeddah, 21589 Saudi Arabia; 3 College of Medicine, University of Jeddah, Jeddah, Saudi Arabia

## Abstract

**Objective:**

To examine the association of weight status with level of diabetes knowledge (symptoms and complications) among residents of Jeddah City, Saudi Arabia.

**Methods:**

In a cross-sectional study, a questionnaire assessing sociodemographic and health characteristics and knowledge about diabetes and its symptoms and complications was utilized. Data of 3978 adults, 18 years of age or older, were collected from public mall sites in Jeddah city and surrounding areas. Participants were divided into three tertiles based on their knowledge scores. Weight and height were measured following standardized procedures, and body weight categories were defined based on body mass index (BMI). The association between weight status and tertiles of diabetes knowledge was examined using multinomial logistic regression analysis.

**Results:**

Compared to normal-weight participants, participants who were underweight, overweight, or obese, did not differ with regards to knowledge about diabetes symptoms. Adjusted models showed that overweight and obese participants had lower odds of being in the lowest tertile of knowledge about diabetes complications compared to normal-weight participants (OR: 0.71, 95% CI: 0.58–0.86 and OR: 0.64, 95% CI: 0.51–0.79, respectively). With regards to general knowledge about diabetes, the knowledge of participants who were underweight did not differ when compared to normal-weight participants. Overweight and obese participants had lower odds of being in the lowest tertile of general knowledge about diabetes compared to normal-weight participants (OR: 0.78, 95% CI: 0.62–0.97 and OR: 0.60, 95% CI: 0.47–0.76, respectively).

**Conclusions:**

Overweight and obese individuals have better knowledge about diabetes compared to normal-weight individuals. Public health programs need to take into account the level of diabetes knowledge and tailor interventions to aid behavior and lifestyle change.

## Introduction

Diabetes mellitus (DM) continues to be a rising public health concern^1^. An estimated 382 million individuals worldwide had DM in 2013, and numbers are expected to exceed 592 million by 2035^2^. At the individual level, DM leads to poorer quality of life and shorter life expectancy;^3^ individuals with diabetes encounter both short-term acute glycemic control issues, as well as long-term chronic complications, including nephropathy, retinopathy, and cardiovascular and atherosclerotic complications^4^. At the population level, increased prevalence of diabetes and associated complications lead to vast medical spending and economic burden^5,6^.

Patterns of increasing prevalence of diabetes is observed in both developed and developing countries^7^. Owing to rapidly changing societal norms, economic growth, and the nutrition transition, Saudi Arabia has experienced a startling increase in obesity and diabetes rates over the past three decades ^8–10^. Latest data suggest that the prevalence of obesity and diabetes in Saudi Arabia is around 30%^11,12^.

Awareness and level of knowledge about aspects of DM; the disease’s symptoms and complications, are important determinants of early detection and improved glycemic control and quality of life^13^. Although it is well-established that individuals with DM can improve disease outcomes and reduce complication risk by taking precautionary and treatment measures^14^, many patients only become aware that they have diabetes after the disease progresses and an associated complication develops^15^. Therefore, adequate knowledge of diabetes’s symptoms and complications are vital for disease control and improvement of outcomes.

Numerous public health programs have been conducted for DM prevention and control, most incorporated means to increase individuals’ awareness about the disease. However, there are inadequate data that describe knowledge level about DM symptoms and complications and how they relate to sociodemographic and health characteristics. Specifically, although some studies reported associations of DM knowledge level with sex, education, and occupation in a Saudi sample^16^, to our knowledge, none evaluated the relationship of diabetes knowledge with nationality, income, marital status, and medical history. Furthermore, we were unable to identify any studies that described the level of knowledge of DM symptoms and complications among obese individuals, a group at high risk for developing the disease^17^.

Identifying and understanding patterns of knowledge among communities before introducing prevention and intervention programs is essential in order to effectively design and target evidence-based programs that are informed by community needs^18^. Assessing the level of knowledge among individuals based on their weight status can aid in accurately defining goals and objectives of public health programs for diabetes prevention, especially among overweight and obese individuals. The present study aimed to examine the association of sociodemographic and health characteristics, including the presence of DM and hypertension (HTN), with level of diabetes knowledge (symptoms and complications) in residents of Jeddah city, Saudi Arabia. We also examined the association between weight status and level of diabetes knowledge (symptoms and complications) among residents of Jeddah city, Saudi Arabia. The outcomes of this study can be used to design, implement, and evaluate intervention programs according to community needs and level of awareness.

## Methods

### Sample

The sample included 3978 participants who were recruited from public malls in Jeddah city and surrounding areas, including Makkah and small villages, during a series of public health campaigns conducted over a period of three months. Inclusion criteria included that the participant is 18 years of age or older, that he/she is a fluent Arabic speaker and a resident of Saudi Arabia. Female participants who were pregnant at the time of data collection were excluded. Fifty-three participants who had missing data were excluded from the dataset. The final sample included in the analysis (*n* = 3925) did not differ from those not included (*n* = 53) with regard to sex, nationality, and educational level. This study was approved by the Unit of Biomedical Ethics at King Abdulaziz University Hospital and confidentiality was maintained as data remained anonymous for all participants.

### Measures

Participants completed a revised questionnaire^16,19^, which consisted of several questions related to sociodemographic and health characteristics and knowledge about diabetes and its symptoms and complications. Due to high prevalence of low literacy, research assistants read the questions and response options aloud from a tablet, then entered participants’ answers. The questionnaire was completed prior to delivery of any information related to diabetes, and consent of participants was obtained by the research assistants.

### Study outcome: diabetes knowledge

Knowledge about DM symptoms was assessed using questions related to frequency of urination, weight gain, poor vision, increased body temperature, excessive thirst, back pain, and tiredness. Each correct answer was scored as 1; responses to irrelevant or false symptoms, such as decreased urination, weight gain, increased body temperature, and lower back pain were reverse-coded to reflect the correct answers (scored out of 7). Knowledge about DM complications was assessed using questions related to risk of cancer (reverse-coded), stroke, vision loss, neurological disorder, renal problems, foot ulcers, and amputation (scored out of 7). An additional question on whether a diabetic patient could avoid complications by adhering to treatment was also included (scored out of 1). Thus, a score for general knowledge about DM was calculated based on the three scores: (1) knowledge about DM symptoms, (2) knowledge about DM complications, and (3) knowledge about effect of adherence to treatment on DM complications (scored out of 15).

In order to better assess characteristics associated with knowledge level, participants were divided into tertiles based on their score on DM symptoms knowledge, DM complications knowledge, and DM general knowledge. The lowest tertile of each type of knowledge is represented by tertile 1, middle tertile represented by tertile 2, and highest tertile represented by tertile 3.

### Primary predictors: sociodemographic, health characteristics, and weight status

Participants reported information regarding their sex (1 = male, 2 = female), nationality (Non-Saudi = 0, Saudi = 1), educational level (0 = illiterate, 1 = elementary school, 2 = middle school, 3 = high school, 4 = 2-year diploma, 5 = bachelor’s degree, 6 = graduate degree) (later collapsed into only 5 categories), total monthly income (1 = less than 5,000 SR per month, 2 = 5,000 to less than 10,000 per month, 3 = 10,000 to less than 15,000 per month, 4 = 15,000 to less than 20,000 per month, 5 = 20,000 or more per month), marital status (0 = single, 1 = married, 2 = widowed, 3 = divorced), current active smoker (0 = no, 1 = yes), history of diabetes (0 = no, 1 = yes) (participants were asked if they were ever diagnosed with any type of diabetes), and history of hypertension (0 = no, 1 = yes) (participants were asked if they were ever diagnosed with hypertension).

Participants’ weight and height were measured by trained research assistants. Body-mass-index (BMI) was calculated by dividing weight in kg by height in meter squared, and weight status categories were defined as: 1 = underweight (BMI < 18.5 kg/m^2^), 2 = normal weight (BMI = 18.5–24.9 kg/m^2^), 3 = overweight (BMI = 25–29.9 kg/m^2^), and 4 = obese (BMI ≥ 30 kg/m^2^)^20^.

### Statistical analysis

Power calculations indicated that the sample size was sufficient to detect the difference in DM knowledge across the weight status groups with a power of 95% (two-sided), estimated effect size of 0.32, and confidence level of 99%. SPSS version 24.0 was utilized for data analysis. Descriptive statistics were conducted to assess characteristics of the study sample. Chi-square statistics was used to examine the association of sociodemographic and health characteristics variables with tertiles of diabetes knowledge. Multinomial logistic regression was used to estimate the Odds ratios (ORs) and associated 95% confidence intervals (95% CI) in order to examine the association between weight status and tertiles of diabetes knowledge. In each of the regression models, the highest tertile (tertile 3) was set as the reference outcome category, and the normal-weight category was set as the reference predictor category. We first ran unadjusted models, then the ORs were further evaluated after adjusting for potential confounders. For all analyses conducted, two-sided tests were used; a probability value of less than 0.05 was considered statistically significant.

## Results

### Sample characteristics

Approximately 56% of the study sample were female and the majority were Saudi (76.4%) (Table 1). About half of the participants (52.2%) had completed a high school degree or less. Nearly one-third of the sample (36.9%) were considered low-income (i.e., total monthly income < 5000 SR); and about half (54.4%) of the sample were married. Furthermore, about a quarter of the sample reported that they were smokers (26.3%). The prevalence of obesity, diabetes, and hypertension was 25.8, 13.3, and 11.2%, respectively.

**Table 1:** Sample characteristics of participants (*n* = 3925)

Characteristics	Category	*N* (%)
Sex	Male	1716 (43.7)
	Female	2209 (56.3)
Nationality	Saudi	2999 (76.4)
	Non-Saudi	926 (23.6)
Educational level	Illiterate	106 (2.7)
	Elementary school	157 (4.0)
	Middle school	325 (8.3)
	High school or diploma	1459 (37.2)
	≥Bachelor’s degree	1878 (47.8)
Monthly income	No income	570 (14.5)
	<5000	878 (22.4)
	5000–<10,000	1188 (30.3)
	10,000–<20,000	1050 (26.8)
	≥20,000	239 (6.1)
Marital status	Single	1545 (39.4)
	Married	2136 (54.4)
	Widowed	98 (2.5)
	Divorced	146 (3.7)
Weight status	Underweight	225 (5.7)
	Normal weight	1462 (37.2)
	Overweight	1224 (31.2)
	Obese	1014 (25.8)
Smoker	Yes	1032 (26.3)
	No	2893 (73.7)
History of diabetes	Yes	523 (13.3)
	No	3402 (86.7)
History of hypertension	Yes	441 (11.2)
	No	3484 (88.8)

### Associations of sociodemographic and health characteristics with diabetes knowledge

The median score of knowledge about DM symptoms was 5 out of a total score of 7 (interquartile range (IQR) 4–6); median score for knowledge about DM complications was 5 out of 7 (IQR 4–6); median score for general knowledge about DM was 11 out of 15 (IQR 10–12).

As shown in Table 2, sex was significantly associated with knowledge about diabetes symptoms, and with general knowledge about diabetes, but not with knowledge about diabetes complications; a higher percentage of females (compared to males) fell into the 2nd and 3rd tertiles of knowledge about diabetes symptoms and general knowledge about diabetes (*P* < 0.05). Additionally, nationality was significantly associated with knowledge about diabetes complications, and with general knowledge about diabetes, but not with knowledge about diabetes symptoms. A higher percentage of Saudis (compared to non-Saudis) fell into the 3rd tertiles of knowledge about diabetes complications and general knowledge about diabetes (*P* < 0.05). Educational level, monthly income, and marital status, were each significantly associated with all types of diabetes knowledge (All *Ps* *<* 0.01). A higher percentage of participants with a bachelor’s degree or higher, those in the highest income category, and those who were divorced, fell into the 3rd tertile of knowledge about diabetes symptoms, knowledge about diabetes complications, and general knowledge about diabetes. Weight status was significantly associated with knowledge about diabetes complications, and with general knowledge about diabetes, but not with knowledge about diabetes symptoms. A higher percentage of obese participants fell into the 3rd tertiles of knowledge about diabetes complications and general knowledge about diabetes (*P* < 0.01).

**Table 2:** Differences in sociodemographic and health characteristics among tertiles of diabetes knowledge (*n* = 3925)

Characteristics	DM symptoms knowledge			*P* value	DM complications knowledge			*P* value	DM general knowledge			*P* value
	Tertile I	Tertile II	Tertile III		Tertile I	Tertile II	Tertile III		Tertile I	Tertile II	Tertile III	
Sex												
Male	1112 (64.8)	406 (23.7)	198 (11.5)	0.03	558 (34.3)	647 (37.7)	481 (28.0)	0.48	757 (44.1)	661 (38.5)	298 (17.4)	<0.01
Female	1344 (60.8)	593 (26.8)	272 (12.3)		740 (33.5)	811 (36.7)	658 (29.8)		846 (38.3)	962 (43.5)	401 (18.2)	
Nationality												
Saudi	1846 (61.6)	784 (26.1)	369 (12.3)	0.06	971 (32.4)	1104 (36.8)	924 (30.8)	<0.01	1182 (39.4)	1249 (41.6)	568 (18.9)	<0.01
Non-Saudi	610 (65.9)	215 (23.2)	101 (10.9)		357 (38.6)	354 (38.2)	215 (23.2)		421 (45.5)	374 (40.4)	131 (14.1)	
Educational level												
Illiterate	82 (77.4)	18 (17.0)	6 (5.70)	<0.01	40 (37.7)	34 (32.1)	32 (30.2)	<0.01	62 (58.5)	35 (33.0)	9 (8.5)	<0.01
Elementary school	119 (75.8)	29 (18.5)	9 (5.7)		53 (33.8)	65 (41.4)	39 (24.8)		92 (58.6)	49 (31.2)	16 (10.2)	
Middle school	220 (67.7)	80 (24.6)	25 (7.7)		139 (42.8)	104 (32.0)	82 (25.2)		165 (50.8)	117 (36.0)	43 (13.2)	
High school or diploma	979 (67.1)	333 (22.8)	147 (10.1)		569 (39.0)	553 (37.9)	337 (23.1)		681 (46.7)	577 (39.5)	201 (13.8)	
≥Bachelor’s degree	1056 (56.2)	539 (28.7)	283 (15.1)		527 (28.1)	702 (37.4)	649 (34.6)		603 (32.1)	845 (45.0)	430 (22.9)	
Monthly income												
No income	384 (67.4)	132 (23.2)	54 (9.5)	<0.01	226 (39.6)	198 (43.7)	146 (25.6)	<0.01	268 (47.0)	228 (40.0)	74 (13.0)	<0.01
<5000	600 (68.3)	210 (23.9)	68 (7.7)		349 (39.7)	328 (37.4)	201 (22.90		420 (47.8)	357 (40.7)	101 (11.5)	
5000–<10,000	751 (63.2)	294 (24.7)	143 (12.0)		390 (32.8)	459 (38.6)	339 (28.5)		472 (39.7)	515 (43.4)	201 (16.9)	
10,000–<20,000	593 (56.5)	296 (28.2)	161 (15.3)		305 (29.0)	387 (36.9)	358 (34.1)		368 (35.0)	431 (41.0)	251 (23.9)	
≥20,000	128 (53.6)	67 (28.0)	44 (18.4)		58 (24.3)	86 (36.0)	95 (39.7)		75 (31.4)	92 (38.5)	72 (30.1)	
Marital status												
Single	1017 (65.8)	374 (24.2)	154 (10.0)	<0.01	675 (43.7)	505 (32.7)	365 (23.6)	<0.01	747 (48.3)	578 (37.4)	220 (14.2)	<0.01
Married	1280 (59.9)	566 (26.5)	290 (13.6)		567 (26.5)	870 (40.7)	699 (32.7)		743 (34.8)	958 (44.9)	435 (20.4)	
Widowed	75 (76.5)	17 (17.3)	6 (6.1)		37 (37.8)	37 (37.8)	24 (24.5)		52 (53.1)	35 (35.7)	11 (11.2)	
Divorced	84 (57.5)	42 (28.8)	20 (13.7)		49 (33.6)	46 (31.5)	51 (34.9)		61 (41.8)	52 (35.6)	33 (22.6)	
Weight status												
Underweight	145 (64.4)	60 (26.7)	20 (8.9)	0.35	114 (50.7)	65 (28.9)	46 (20.4)	<0.01	113 (50.2)	85 (37.8)	27 (12.0)	<0.01
Normal weight	919 (62.9)	368 (25.2)	175 (12.0)		559 (38.2)	541 (37.0)	362 (24.8)		662 (45.3)	572 (39.1)	228 (15.6)	
Overweight	785 (64.1)	296 (24.2)	143 (11.7)		377 (30.8)	452 (36.9)	395 (32.3)		471 (38.5)	524 (42.8)	229 (18.7)	
Obese	607 (59.9)	275 (27.1)	132 (13.0)		278 (27.4)	400 (39.4)	336 (33.1)		357 (35.2)	442 (43.6)	215 (21.2)	
Smoker												
Yes	681 (66.0)	224 (21.7)	127 (12.3)	0.01	338 (32.8)	407 (39.4)	287 (27.8)	0.21	447 (43.3)	409 (39.6)	176 (17.1)	0.17
No	1775 (61.4)	775 (26.8)	343 (11.9)		990 (34.2)	1051 (36.3)	852 (29.5)		1156 (40.0)	1214 (42.0)	523 (18.1)	
History of diabetes												
Yes	360 (68.8)	106 (20.3)	57 (10.9)	0.01	112 (21.4)	217 (41.5)	194 (37.1)	<0.01	211 (40.3)	208 (39.8)	104 (19.9)	0.39
No	2096 (61.6)	893 (26.2)	413 (12.1)		1216 (35.7)	1241 (36.5)	945 (27.8)		1392 (40.9)	1415 (41.6)	595 (17.5)	
History of hypertension												
Yes	306 (69.4)	96 (21.8)	39 (8.8)	0.01	114 (25.9)	175 (39.7)	152 (34.5)	<0.01	181 (41.0)	183 (41.5)	77 (17.5)	0.98
No	2150 (61.7)	903 (25.9)	431 (12.4)		1214 (34.8)	1283 (36.8)	987 (28.3)		1422 (40.8)	1440 (41.3)	622 (17.9)	

### Associations between weight status and diabetes knowledge

#### Knowledge about DM symptoms

As shown in Table 3, compared to normal-weight participants, participants who were underweight, overweight, or obese, did not differ with regards to knowledge about diabetes symptoms. These results did not change after adjusting for various variables (All *Ps* *>* 0.05).

**Table 3:** Multinomial logistic regression of weight status on diabetes knowledge

	Odds ratios^26^ and 95 percent confidence intervals (CIs)					
	DM symptoms knowledge		DM complications knowledge		General DM knowledge	
	Tertile I to reference category	Tertile II to reference category	Tertile I to reference category	Tertile II to reference category	Tertile I to reference category	Tertile II to reference category
Model 1						
Weigh status						
Underweight	1.38 (0.84–2.27)	1.43 (0.83–2.44)	1.61 (1.11–2.32)*	0.95 (0.63–1.41)	1.44 (0.92–2.26)	1.26 (0.79–1.99)
Overweight	1.05 (0.82–1.33)	0.98 (0.75–1.29)	0.62 (0.51–0.75)*	0.77 (0.63–0.93)*	0.71 (0.57–0.88)*	0.91 (0.73–1.14)
Obese	0.88 (0.68–1.12)	0.99 (0.75–1.30)	0.54 (0.44–0.66)*	0.80 (0.65–0.97)*	0.57 (0.46–0.72)*	0.82 (0.66–1.03)
Normal (reference)	1	1	1	1	1	1
Model 2						
Weigh status						
Underweight	1.42 (0.86–2.33)	1.42 (0.83–2.43)	1.67 (1.51–2.41)*	0.97 (0.65–1.45)	1.51 (0.97–2.37)	1.25 (0.79–1.98)
Overweight	1.03 (0.81–1.31)	0.99 (0.76–1.30)	0.61 (0.50–0.74)*	0.76 (0.62–0.92)*	0.69 (0.55–0.86)*	0.92 (0.74–1.15)
Obese	0.87 (0.68–1.12)	0.99 (0.76–1.31)	0.54 (0.44–0.66)*	0.80 (0.66–0.97)*	0.57 (0.45–0.72)*	0.83 (0.66–1.04)
Normal (reference)	1	1	1	1	1	1
Model 3						
Weigh status						
Underweight	1.29 (0.78–2.13)	1.35 (0.79–2.32)	1.48 (1.02–2.15)*	0.94 (0.63–1.41)	1.31 (0.85–2.12)	1.18 (0.74–1.87)
Overweight	1.13 (0.88–1.45)	1.04 (0.79–1.37)	0.68 (0.55–0.83)*	0.78 (0.64–0.94)*	0.77 (0.61–0.96)*	0.98 (0.78–1.22)
Obese	0.90 (0.70–1.17)	1.02 (0.77–1.35)	0.58 (0.47–0.73)*	0.81(0.66–0.99)*	0.58 (0.46–0.74)*	0.86 (0.68–1.08)
Normal (reference)	1	1	1	1	1	1
Model 4						
Weigh status						
Underweight	1.29 (0.78–2.14)	1.34 (0.78–2.30)	1.45 (1.00–2.12)	0.94 (0.63–1.41)	1.34 (0.85–2.12)	1.18 (0.74–1.87)
Overweight	1.11 (0.86–1.42)	1.04 (0.79–1.37)	0.71 (0.58–0.86)*	0.79 (0.65–0.96)*	0.78 (0.62–0.97)*	0.98 (0.79–1.23)
Obese	0.87 (0.67–1.12)	1.01 (0.76–1.35)	0.64 (0.51–0.79)*	0.84 (0.68–1.03)	0.60 (0.47–0.76)*	0.87 (0.69–1.10)
Normal (reference)	1	1	1	1	1	1

Model 1: unadjusted

Model 2: Adjusted for sex and nationality

Model 3: Adjusted for sex, nationality, educational level, marital status, and income

Model 4: Adjusted for sex, nationality, educational level, marital status, income, smoking, history of diabetes, and history of hypertension

*P value < 0.05

#### Knowledge about DM complications

Compared to normal-weight participants, underweight participants had higher odds of being in the lowest tertile of knowledge about DM complications (OR: 1.61, 95% confidence interval (CI): 1.11–2.32) (Model 1). Adjusting for sex and nationality did not weaken this association (OR: 1.67, 95% CI: 1.51–2.41) (Model 2). However, the OR for underweight participants was diminished when educational level, marital status, and income were simultaneously added (OR: 1.48, CI: 1.02–2.15) (Model 3). The association disappeared when smoking, history of diabetes, and history of hypertension were added (OR: 1.45, CI: 1.00–2.12) (Model 4) (Separate analyses revealed effect to be attributed to adding “history of diabetes” to the model).

Overweight participants had lower odds of being in the lowest tertile of knowledge about DM complications compared to normal-weight participants (OR: 0.62, 95% CI: (0.51–0.75) (Model 1). The association remained the same after adjusting for sex and nationality (OR: 0.61, 95% CI: 0.50–0.74) (Model 2), but a slight increase in the OR was observed after adjusting for educational level, marital status, and income (less than 10% change in beta coefficient) (OR: 0.68, 95% CI: 0.55–0.83) (Model 3). After adjusting for smoking, history of diabetes, and history of hypertension, the OR for overweight participants increased significantly (i.e., more than 10% change in beta coefficient) (OR: 0.71, 95% CI: 0.58–0.86) (Model 4). (Separate analyses revealed effect to be attributed to adding “history of diabetes” to the model).

Obese participants had lower odds of being in the lowest tertile of knowledge about DM complications compared to normal-weight participants (OR:0.54, 95% CI: 0.44–0.66) (Model 1). The association remained the same after adjusting for sex and nationality (OR: 0.54, 95% CI: 0.44–0.66) (Model 2). However, the OR for obese participants slightly increased when educational level, marital status, and income were simultaneously added (0.58 (0.47–0.73) (Model 3). The OR for obese participants significantly increased when smoking, history of diabetes, and history of hypertension were added (i.e., more than 10% change in beta coefficient) (OR: 0.64, CI: 0.51–0.79) (Model 4) (Separate analyses revealed effect to be attributed to adding “history of diabetes” to the model).

#### General knowledge about DM

Compared to normal-weight participants, participants who were underweight did not differ with regards to general knowledge about diabetes. These results did not change after adjusting for various variables (All *Ps* *>* 0.05). Overweight participants had lower odds of being in the lowest tertile of general knowledge about DM compared to normal-weight participants (OR: 0.71 (95% CI: 0.57–0.88) (Model 1). The association did not meaningfully change after adjusting for sex and nationality (OR: 0.69, 95% CI: (0.55–0.86) (Model 2). A slight increase in the OR was observed after adjusting for educational level, marital status, and income (no more than 10% change in beta coefficient) (OR: 0.77, 95% CI: 0.61–0.96) (Model 3), and after adjusting for smoking, history of diabetes, and history of hypertension (OR: 0.78, 95% CI 0.62–0.97) (Model 4).

Obese participants had lower odds of being in the lowest tertile of general knowledge about DM compared to normal-weight participants (OR: 0.57, 95% CI: 0.46–0.72) (Model 1). The association remained the same after adjusting for sex and nationality (OR: 0.57, 95%: 0.45–0.72) (Model 2). When educational level, marital status, and income were simultaneously added, the OR did not meaningfully change (OR: 0.58, 95% CI: 0.46–0.72) (Model 3). The OR for obese participants did not meaningfully change when smoking, history of diabetes, and history of hypertension were added to the model (OR: 0.60, 95% CI: 0.47–0.76) (Model 4).

## Discussion

We found that sociodemographic, health and weight status characteristics were each associated with level of knowledge about diabetes in a sample of Saudi residents. In the present study, we sought to define and assess the level of knowledge by categorizing knowledge into tertiles rather than subjectively choosing a cutoff for adequate knowledge vs. not. Therefore, assessing the level of knowledge about DM symptoms revealed that the majority of participants (62.6%) were in the lowest tertile of knowledge. This is contrary to findings of a previous study conducted in Al-Qassim, Saudi Arabia, which reported a high percentage (80%) of “good knowledge about DM symptoms”, where “good knowledge” was defined as mean score of ≥10 out of 15^16^. Furthermore, we found that around one third of the sample fell into each of the 3 tertiles with regards to knowledge about DM complications (33.8% in tertile 1, 37.1% in tertile 2, and 29% in tertile 3) indicating that the majority of participants (66.1%) had low to moderate knowledge about the complications of DM. This is consistent with findings of Al-Qassim study, which reported concerning levels of unawareness about complications^16^. Similarly, the majority of participants in our sample fell into the lowest (tertile 1) and middle (tertile 2) tertiles with regards to overall knowledge about DM (40.8 and 41.4%, respectively). These findings are alarming and reflect the urgent need to increase the public’s knowledge and awareness of diabetes. Indeed, lack of knowledge about diabetes symptoms and complications may result in delayed detection, diagnosis, and management. Occasionally, due to the delayed diagnosis, complications that have already occurred lead patients to believe that the complications were precipitated by the treatment regimen and not the underlying process itself. This misconception is propagated throughout the community causing further delays in seeking help. Therefore, community initiatives are needed for educating the public about DM symptoms, which is critical for early detection and improved quality of life^13^. Efforts are also needed for increasing awareness about DM complications in order to optimize treatment outcomes, as suggested by previous studies^19,21^.

The results of the present study indicated a higher level of knowledge about DM symptoms and general knowledge among females compared to males, while no significant difference between males and females was observed with regards to DM complications. This is in contrast to national and international findings, which showed a greater level of knowledge about DM among males compared to females^16,19^.

When compared to non-Saudis, Saudi participants appeared to have better knowledge about diabetes complications and general knowledge about diabetes. This could be related partially to the educational level attained by the participants, as 86.5% of Saudi participants were at least high school graduates compared to 80% of the non-Saudi participants. Additionally, Saudi participants had higher income compared to non-Saudis, such that 37% of Saudis reported to have a total monthly income of >10,000 SR compared to only 15.7% of non-Saudis. These results are in line with findings from previous studies, which also reported a positive association between education and income level and DM knowledge^16,22^.

In order to assess the association of a previous diagnosis of DM and HTN with knowledge about DM, we included participants with a history of DM and HTN in our sample. HTN is a common comorbid condition of which the risk doubles among diabetic patients compared to non-diabetics^23^. Interestingly, participants with a history of DM and/or HTN were found to have significantly lower knowledge about DM symptoms compared to participants with no history of DM/HTN, while their knowledge about DM complications was greater. Thus, given discrepancies in level of knowledge between diabetics and non-diabetics, including both groups in the study sample when examining knowledge about DM symptoms and complications may be relevant in designing public health programs and community campaigns. This can aid in understanding knowledge deficits among each group, and programs can be designed and targeted accordingly.

In the present study, we also aimed to assess the level of knowledge about DM symptoms, complications, and general knowledge among participants in different weight status categories. Upon stratification by BMI categories, the level of knowledge about DM symptoms among participants who were underweight, overweight, and obese did not significantly differ when compared to participants who were normal-weight. However, obese participants were found to have greater knowledge about DM and its complications compared to non-obese participants. When compared to normal-weight participants, overweight and obese participants were found to have lower likelihood of having poorer knowledge about DM complications and general DM knowledge.

Although both practitioners and community initiatives may target obese individuals in clinics and awareness programs due to their increased risk for developing the disease^17^, our study highlights the need to specifically focus on motivating obese individuals to implement lifestyle changes and tailor messages to their readiness for change^24^. In addition, public health professionals and educators may need to assess obese individuals’ attitudes toward the health problem, and identify their perceived barriers to behavior change. Indeed, a previous study that evaluated an intervention program based on the Health Belief Model found evidence of effectiveness of the program among diabetic patients^25^. Hence, the goal of education should be to facilitate the adoption of behaviors conducive to health and well-being, with focus shifted from simply increasing knowledge to addressing factors that serve as mediators for behavior change.

This study has several limitations. First, in order to reduce respondents’ burden and increase response rate, we did not distinguish between types of DM and how associations with knowledge may differ. Future studies may focus on comparing knowledge among participants with different types of DM. Furthermore, there are other factors that may be associated with DM knowledge that were not taken into account in this study, such as having a family member diagnosed with DM and frequency of exposure to awareness campaigns. Additional studies are needed in order to identify other factors associated with DM knowledge. Specifically, studies that include qualitative data collection and in-depth interviewing may be beneficial. This study also has several strengths. To our knowledge, this is the first study that quantifies DM knowledge by categorizing it into tertiles, rather than using a subjective cut-off for “poor” vs. “good” knowledge. This study was also the first to quantify level of knowledge about DM based on weight status, considering obesity as a critical DM risk factor. Furthermore, our sample size is large, increasing our statistical power and reducing type 2 error.

Findings from the present study can aid in recognizing gaps in public health programs, awareness campaigns, and educational efforts by health professionals. Strategies can be designed to specifically target individuals who have shown poorer knowledge of DM, such as male and non-Saudi members of the community. Furthermore, programs can be specifically tailored for overweight and obese individuals with a focus on addressing barriers and readiness for change, in addition to other psychosocial constructs.

In conclusion, continual aspiration to increase knowledge about DM, specifically DM complications, is needed among Saudi residents. Overweight and obese individuals have better knowledge about DM compared to normal-weight individuals, and tailored efforts to aid behavior and lifestyle change is needed. With the emerging epidemic of diabetes, further research to identify barriers to adopt healthy behaviors by high risk groups and to increase public knowledge and awareness of DM is essential.
